# Anti-inflammatory and antioxidant activity of high concentrations of hydrogen in the lung diseases: a systematic review and meta-analysis

**DOI:** 10.3389/fimmu.2024.1444958

**Published:** 2024-08-15

**Authors:** Kang Xiao, Jianwei Liu, Yuxin Sun, Shangya Chen, Jiazi Ma, Mao Cao, Yong Yang, Zhifeng Pan, Peng Li, Zhongjun Du

**Affiliations:** ^1^ Shandong Academy of Occupational Health and Occupational Medicine, Shandong First Medical University & Shandong Academy of Medical Sciences, Ji’nan, Shandong, China; ^2^ School of Basic Medicine and Clinical Pharmacy, China Pharmaceutical University, Nanjing, Jiangsu, China; ^3^ Public Health Monitoring and Evaluation Institute of Shandong Provincial Center for Disease Control and Prevention, Ji’nan, Shandong, China

**Keywords:** anti-inflammatory, antioxidant, high-concentration hydrogen, meta-analysis, respiratory system, systematic review

## Abstract

As a small molecule, hydrogen is colorless, odorless and lightest. Many studies conducted that hydrogen can protect almost every organ, including the brain, heart muscle, liver, small intestine, and lungs. To verify whether high concentrations of hydrogen (HCH) has anti-inflammatory and antioxidant activities on respiratory system, we product a systematic review and meta-analysis. We investigated MEDLINE-PubMed, Cochrane Library, ScienceDirect, Wiley and SpringerLink database and selected *in vivo* studies related to the anti-inflammatory or antioxidant effects of HCH in the lung diseases which were published until September 2023. We firstly identified 437 studies and only 12 met the inclusion criteria. They all conducted in rodents. The results showed that HCH had a positive effect on the reduction of tumor necrosis factor alpha (TNF-α), interleukin (IL)-1β, IL-4, IL-8, malondialdehyde (MDA), superoxide dismutase (SOD) and reactive oxygen species (ROS); but there is no effect on IL-6, we speculated that may contribute to the test results for different body fluids and at different points in time. This meta-analysis discovered the protective effects on inflammation and oxidative stress, but whether there exists more effects on reduction of inflammatory and oxidant mediators needs to be further elucidated.

## Background

By now, the incidence of respiratory diseases is increasing, and its mortality rate is among the top three in the world. It also imposes a huge economic burden. In addition, it has an emotional impact on patients (1).. Therefore, there needs a more efficient and economical method to save patients’ survival and quality of life. Many researchers believe that pulmonary inhalation may be a more direct and effective way with fewer side effects. Many similar studies are under way. Hydrogen, for example, is a treatment that is inhaled directly into lungs.

Hydrogen (H_2_), a diatomic gas composed of two hydrogen atoms connected by covalent bonds, is produced by the intestinal bacteria of mammals; H_2_ is colorless and odorless and is a stable neutral molecule (2). In 2007, Ohsawa et al (3) reported that H_2_ can react with cytotoxic oxygen free radicals by reacting with hydroxyl free radicals (•OH) in cultured cells. H_2_ does not react with •O^2-^, H_2_O_2_ or NO. Due to its potential ability to anti oxidative stress, inflammation, and apoptosis, H_2_ is emerging as the fourth gas signaling molecule in the body (4). Generally, hydrogen concentrations between 4% and 75% will not increase, and this paper defines hydrogen concentrations above 4% as high-concentration hydrogen (HCH). A systematic review by Yuan et al. (5) reported its potential protective effects on ischemia/reperfusion injury in multiple organs, neurodegenerative diseases, bone and joint diseases, and respiratory diseases.

The commonly used hydrogen administration methods include direct inhalation of hydrogen, injection of hydrogen-rich water and oral hydrogen-rich water (6). This paper mainly explored the therapeutic effect of hydrogen inhalation on respiratory diseases. In 1975, American scholar Dole et al. (7) reported in Science that inhaling hydrogen at 8 atmospheres for 14 consecutive days could significantly reduce the size of skin cancer tumors in mice; this was the first study in human history to determine the medical effect of hydrogen. In 2007, Wood et al (8) evaluated hydrogen as a cytoprotective therapy for ischemia-reperfusion injury and stroke, calling it a selective antioxidant with explosive potential, and this effect has also been confirmed in human experiments (9). At first, most experiments explored the therapeutic effects of low concentrations of hydrogen, but considering the actual concentration of hydrogen inhaled in the body, a higher concentration of hydrogen was derived.

Clinically, Chen et al. (10) reported that inhaling 67% hydrogen can alleviate the disease progression of non-small cell lung cancer; Zheng et al. (11) found that hydrogen therapy can treat acute episodes of chronic obstructive pulmonary disease (COPD); Akagi et al. (12) found that hydrogen can improve the prognosis of advanced colorectal cancer patients; Zeng et al. (13) reported that in the treatment of COVID-19, a mixture of hydrogen and oxygen can improve patients’ percutaneous arterial oxygen saturation (SpO_2_) and shorten the length of hospital stay. Some animal experiments have shown that a high concentration of hydrogen can reduce the secretion of inflammatory factors, possibly through a variety of signaling pathways, such as nuclear factor-kappa B (NF-κb), and can reduce the content of reactive oxygen species (ROS) and some oxidation products.

These studies verified the therapeutic effects of HCH. In order to further evaluate its anti-inflammatory and antioxidant capacity in respiratory diseases, we demonstrated this through this systematic review and meta-analysis.

## Methods

We conducted this study following the Preferred Reporting Items for Systematic Reviews and Meta-Analyses (PRISMA) statement (14) and Cochrane Manual.

### Eligibility criteria

Our criteria for inclusion are: (a) the experimental model was an animal model with lung disease; (b) the intervention was treatment inhalation with high concentration hydrogen alone; (c) the results are an indicator of anti-inflammatory or antioxidant outcomes in the treatment of lung disease; and (d) the type of study was experimental.

All the retrieved titles, abstracts, and full texts were read and screened independently by at least two researchers. If a disagreement arises, it is discussed with reference to the inclusion exclusion criteria. The inclusion criteria were as follows: animal studies; suffers from respiratory problems; high concentration hydrogen inhalation was used alone; and anti-inflammatory or antioxidant outcome measures were used. The exclusion criteria were as follows: articles that do not meet the inclusion criteria, review articles, meta-analyses, abstracts, conference proceedings, editorials/letters, and case report.

### Search strategy

Five databases were used to search for papers that met the criteria of the study: the National Library of Medicine (MEDLINE-PubMed), Cochrane Library, ScienceDirect, Wiley and SpringerLink databases. Different combinations of the following keywords were used: “hydrogen,” “respiratory tract diseases,” “respiratory system,” “lung injury,” “pulmonary” and “trachea”.

The search strategy is as follows: (hydrogen gas) AND (respiratory system disease or lung disease or pulmonary disease or trachea disease). In addition, we checked the references in the article to make sure there were no potential missing articles.

The databases were searched for studies published until September 2023. This retrieval strategy was used to search for the anti-inflammatory and antioxidant effects of high concentration hydrogen in animal models of respiratory diseases. After reading the retrieved literature, we investigated the relevant references and included the relevant articles in the study. We did not contact the original author when there was data in the article that was not available, nor did we cite data from unpublished articles.

### Data collection process and study risk of bias assessment

The data were extracted by one researcher according to **Table 1** and examined by another researcher. The data to be extracted were as follows: study design, animal model studied, methodological characteristics of high concentrations of hydrogen, respiratory injury studied, markers evaluated, main results, conclusions.

**Table 1:** Description of the main aspects of the studies included in the systematic review.

Authors, year, country	Study design	Model	Methodological characteristics of hydrogen gas inhalation	Lesion studied (respiratory system)	Assessed markers	Main results	Conclusion
du et al(2022)China (15)	Experimental	Mouse	66.7% H2 for 2 h after intratracheal instillation of LPS	ALI	pulmonary pathological changes; IL-1β, IL-8 and TNF-α; the mRNA expression of ICAM-1 and VCAM-1 in the lung tissue; lung MDA level; vascular and cellular permeability; NF-κB/CAT pathway in a sirt1-dependent manner	HCH alleviated lung pathological changes and pulmonary edema, and reduced the BALF levels of IL-1β and TNF-α; increased the levels of ICAM-1, VCAM-1 and MDA; improved vascular and cellular permeability; downregulated NF-κB expression and upregulated CAT expression.	hydrogen suppressed inflammatory response and oxidative stress mediated by NF-κB and CAT in a sirt-1 dependent manner
feng et al(2019)China (16)	Experimental, randomized with control group	Rats	67% H2 for 2 h after CAPs exposure	ALI	lung mechanics and pulmonary function; mucus secretion and MUC5AC expression; MDA; 8-iso-PGF2α; H&E staining; TNF-α, IL-1β and IL-8; AhR protein	HCH improved lung mechanics and pulmonary function; inhibited mucus hypersecretion and MUC5AC expression; decreased the levels of MDA and 8-iso-PG; decreased inflammatory scores; decreased the BALF levels of IL-1β, IL-8 and TNF-α; increased the expression of AhR protein.	hydrogen could ameliorate pulmonary dysfunction, airway mucus hypersecretion, oxidation damage, and inflammation response. Additionally, hydrogen alleviates lung injury possibly through AhR-dependent mechanisms
**huang et al.** (2019) **China** (17)	Experimental, randomized with control group	Mouse	42% H2 for twice a day (2 h per time) kept for 7 days	Asthma	airway responsiveness, histopathologic examination, serum total IgE, levels of IL-4, IL-5 and IL-13 in BALF, the percentage of TH1/TH2/TH17 cells, the phagocytic ability of alveolar macrophages, MDA level, SOD activity, NF-κB activation, Nrf2 and HO-1 expression	HCH decreased airway hyperresponsiveness, diminished OVA-induced TH2 responses, decreased the level of IL-4 in BALF and the level of IgE in serum, increased alveolar macrophage phagocytosis, decreased MDA level and increased SOD activity, inhibited OVA-induced NF-κB activation, activated Nrf2 and HO-1 expression	hydrogen gas inhalation enhanced alveolar macrophage phagocytosis in OVA-induced asthmatic mice, which may be associated with the antioxidant effects of hydrogen gas and the activation of the Nrf2 pathway.
**li et al.** (2022) **China** (18)	Experimental, randomized with control group	Rats	42% H2 for 1 h daily after the TBI for 24 h, 48 h, 72	ALI	Arterial blood gas, lung wet/dry ratio, brain edema, histology of brain, histology and lung injury scoring, levels of IL-1β and IL-18, expression of Caspase1, ASC, GSDM-D, Caspase3, BCL-2, and bax,	HCH ameliorates the severity of TBI, improved oxygenation, ameliorates the severity of TBI-induced ALI, reduced IL-1β and IL-18, reduced Caspase-1, GSDM-D and ASC, reduced Caspase-3 and Bax and increased Bcl-2 levels	H2 improves TBI-ALI, and the mechanism may be due to the decrease of both pyroptosis and apoptosis and the alleviation of inflammation.
**lu et al.** (2018) **China** (19)	Experimental, randomized with control group	Mouse	42% H2 for 1 h daily, twice per day for 30 days	COPD	lung function, hematocrit, cell counts in BALF, histological staining, IL-6, TNF-α, Muc5ac and Muc5b in BALF, ERK1/2 and NF-κB expression in lung tissue	HCH improved lung function and hypoxia-induced hematocrit elevation; attenuates emphysema, collagen deposition in the small airway and goblet cell hypertrophy and hyperplasia of airway epithelium; attenuated the high level of total leukocyte number, IL-6, TNF-α, KC, Muc5ac and Muc5b; reduced the levels of ERK1/2 and NF-κB in lung tissue	H2 inhalation could inhibit COPD development in mice, which is associated with reduced ERK1/2 and NF-κB-dependent inflammatory responses.
sun et al(2021)China (20)	Experimental, randomized with control group	Mouse	67% H2 for 1 h At 1 h and 6 h after LPS areosol inhalation	ALI	Histological examination; total cells and PMN in BALF; total protein content and MPO activity; TUNEL apptosis assay; caspase-3 acitivity; TNF-α, IL-1β, IL-6, KC, MIP-1α, MIP-2 and MCP-1; Nrf2 level; ROS levels	HCH significantly downregulated the lung histological score, lung wet/dry weight ratio, improved the lung oxygenation function; reduced the protein concentration, the MPO activity of lung tissue; decreased caspase-3 activity, the number of TUNEL-positive cells, total cell content, polymorphonuclear granulocyte content, the BALF levels of TNF-α, IL-1β, IL-6, the levels of HMGB1, KC, MIP-1α, MIP-2, MCP-1, the level of ROS, improved Nrf2 expression and decreased NF-κB expression.	H2 can effectively alleviate LPS-induced ALI, which may be related to activation of Nrf2 signaling pathway and inhibition of inflammatory response and cell apoptosis mediated by NF-κB.
wang et al(2018)China (21)	Experimental	Mouse	60% H2 for 2 h every day for 4 weeks	Lung cancer	HE staining; the protein expression levels of Ki-67, VEGF and SMC3; the levels of ROS, SOD and pro-inflammatory factors such as IL-1β, IL-8, IL-13 and TNF-α	HCH could reverse the pathological lung tissue into approximately normal, the protein expression of Ki-67, VEGF and SMC3 were all reduced, the ROS level was reduced and SOD level was increased, the levels of IL-1β, IL-8, IL-13 and TNF-α were all reduced, the weights of tumor were reduced.	H2 inhibited the carcinogenesis in lung cancer, and exerted antioxidant and inflammatory roles
**wei et al.** (2023) **China** (22)	Experimental, randomized with control group	Mouse	After creation of the inflammation model, 42% hydrogen inhalation for 1 h, 3 h, 6 h.	ALI	Histological examinations, IL-1α, IL-1β, IL-2, IL-3, IL-4, IL-5, IL-6, IL-9, IL-10, IL-12p40, IL-12p70, IL-13, IL-17, CCL11, CSF3, CSF2, IFN-γ, KC, MCP-1, MIP-1α, MIP-1β, CCL5 and TNF-α; The mRNA levels of MCP-1, MIP-1α, G-CSF, CCL5, and Eotaxin-1	HCH alleviated the pathological inflammatory changes in the tissues; inhibited the secretion of IL-1α, IL-12p40, TNF-α, MCP-1, MIP-1α, MIP-1β, RANTES, and G-CSF at 1 h; decreased MCP-1, MIP-1α, G-CSF, CCCL5 transcription in peritoneal macrophages	hydrogen is potentially inhibitive against inflammation by inhibiting HIF-1α and IL-1α release at early occurrence. The target of the inhibitive LPS-induced-inflammatory action of hydrogen is chemokines in macrophages in the peritoneal cavity.
**yin et al.** (2022) **China** (23)	Experimental, randomized with control group	Mouse	42% hydrogen gas for 72 h after the injection of LPS or saline.	ALI	survival rate; histological examinations; the concentrations of IL-1β, TNF-α, IL-6, and IL-10; MDA and NO levels in lung tissues; TLR4 expressions in lung tissues	HCH improved the survival rate; reduced the MDA and NO concentration; reduced the TNF-α and IL-1β level; prevented the histopathological changes; reduced the expression of TLR4	Hydrogen gas alleviates LPS-induced acute lung injury and infammatory response most likely through the TLR4-NF-κB pathway
**zhang et al(2018)China**	Experimental, randomized with control group	Mouse	200 ml/min; 67% hydrogen for 1 h once a day for 1 week	Asthma	lung resistance; histology and mucus production; inflammatory cells in BALF; IL-4, IL-5, IL-13, TNF-α, IL-6, CXCL15 in BALF and serum; SOD, MDA, GSH, CAT, MPO, and 8-OHdG in lung tissue	HCH decreased lung resistance, reversed the severe inflammatory infiltration and goblet cell hyperplasia, reduced significantly the number of total cells, eosinophils and lymphocytes in BALF, decreased the serum and BALF level of IL-4, IL-13, TNF-α and CXCL15, increased the levels of SOD, GSH, CAT, decreased the levels of MDA, MPO.	Hydrogen gas inhalation improves lung function and protects established airway inflammation in the allergic asthmatic mice model which may be associated with the inhibition of oxidative stress process.
**zhang et al(2021)China** (24)	Experimental, randomized with control group	Mouse	60% H2 for 2 h per day for 2 weeks	Asthma	serum and BALF levels of IL-4, IL-25, IL-33, TSLP and MCP-1, IFN-γ, NF-κB and ST2, E-cadherin, ZO-1, caspase 3 and caspase 9, the population of lineage ILC	Serum and BALF levels of IL-33, IL-4, IL-25, TSLP, and MCP-1, were greatly decreased by H2. Serum and BALF levels of IFN-γ was increased by H2. The expression of NF-κB (p65) and ST2 was decreased by H2. ILC2 population was decreased by H2. E-cadherin and ZO-1 levels in airway tissues was increased by H2 treatment, caspase 3 and caspase 9 were decreased in H2 group, hydrogen gas reduced ICOS+ST2+ cells	Hydrogen treatment reduces allergen-induced asthma due to its anti-inflammatory effects.
**zhao et al(2023)China** (25)	Experimental, randomized with control group	Mouse	4 L/min; H2 was administered by inhalation for 60 min at 1 h and 6 h after the CLP operation.	ALI	the arterial blood PaCO2, PaO2 and pH values, 7-day survival rate, the protein content in BALF, lung wet-to-dry ratio and lung MPO activity, the lung pathological, liver and kidney function, SOD and CAT, 8-iso-PGF2α, HMGB1, the morphology of lung mitochondria, RCR, MMP, mitochondrial respiratory chain complex activities, and expression of fusion and fission proteins	hydrogen improves the 7-day survival rate, decreased the protein content in BALF, lung wet-to-dry ratio and lung MPO activity, reduces acute lung injury as well as liver and kidney injury in sepsis, increased the level of CAT and SOD, decreased the level of 8-iso-PGF2α and HMGB1, compared with the Sham group, mitochondrial dysfunction was alleviated in hydrogen groups.	High concentration hydrogen inhalation can significantly reduce the lung injury in septic mice and improve the mitochondrial dynamic balance due to its antioxidative and anti-inflammatory effects.

ALI, acute lung injury; H2, hydrogen; BALF, bronchoalveolar lavage fluid; CAT, catalase; sirt1, sirtuin-1; ICAM-1, intercellular cell adhesion molecule-1; VCAM-1, Vascular Cell Adhesion Protein 1; CAPs, concentrated ambient particles; MUC5A, mucin 5AC; 8-iso-PGF2α, 8-iso-prostaglandin F2α; AhR, aryl hydrocarbon receptor; PMN, polymorphonuclear neutrophil; TUNEL, TdT-mediated dUTP Nick-End Labeling; KC, keratinocyte-derived chemokine; MIP-1α, macrophage inflammatory protein-1α; MIP-2, macrophage inflammatory protein-2; MCP-1, monocyte chemoattractant protein-1; Nrf2, nuclear factor erythroid-related factor 2; HO-1, heme oxygenase 1; ASC, apoptosis-associated speck-like protein containing CARD;GSDM-D, Gasdermin-D; BCL-2, B-cell lymphoma-2; TBI, traumatic brain injury; ERK, extracellular regulated protein kinases; ROS, reactive oxygen species; HE, hematoxylin and eosin; Ki-67, Antigens Ki67; VEGF, vascular endothelial growth factor; SMC3, structural maintenance of chromosomes protein 3; CXCL, chemokine C-X-C-motif ligand; CCL, chemokine C-C motif ligand; CSF, colony stimulating factor; GSH, L-Glutathione; NO, nitric oxide; TLR, toll like receptor; MPO, myeloperoxidase; 8-OHdG, 8-hydroxy-2 deoxyguanosine; TSLP, thymic stromal lymphopoietin; ST2, tumorigenicity 2 receptor; ZO-1, zona occludens 1; ILC, innate lymphoid cell; HMGB1, high mobility group box 1 protein; RCR, Mitochondrial Respiratory Control Rate; MMP, Mitochondrial membrane potential; IFNy, interferon gamma; IL, interleukin; LPS, lipopolysaccharides; MDA, malondialdehyde; MPO, myeloperoxidase; NF-kB, nuclear factor-kappa B; SOD, superoxide dismutase; TNF-α, alpha tumor necrosis factor.

According to the Cochrane Manual, since we analyzed fewer than 10 articles in each group, we used SYRCLE’s risk of bias (RoB) tool for animal studies to assess the study risk of bias. Each article was evaluated by two different researchers, and if there was any disagreement, it was resolved through negotiation. The risk of bias was rated as low, uncertain, or high. The contents include Selection bias, Performance bias, Detection bias, Attrition bias, Reporting bias and Other bias.

### Synthesis methods

We used standardized mean difference (SMDs) of 95% confidence intervals (CI) to evaluate the treatment effect. If SMD = 0, it indicates no difference, SMD > 0 indicates more occurrence in the experimental group, and SMD < 0 indicates less occurrence in the experimental group. The mean and standard deviation (SD) of the control and treatment groups are obtained by extracting graphs in the article, and the effect size of the target outcome will be calculated. The negative effect size indicated that HCH could effectively reduce inflammatory mediators and markers of oxidative stress, and the positive effect size indicated that HCH could effectively reduce oxidative stress response for superoxide dismutase (SOD).

We used forest maps to graphically represent the effect size and 95% CI. We used Z test to evaluate the overall effect. If P < 0.05, it indicates that there was a significant difference. We used Chi^2^ test to evaluate the heterogeneity of the literature. If I^2^ < 50% or P > 0.1, the heterogeneity is small, and the fixed-effect model is used. If I^2^ ≥ 50% or P ≤ 0.1, it indicates that there is large heterogeneity in the study, and a random-effects model is used. We performed subgroup analyses based on the markers analyzed. If the results of the study did not include numerical values for the target results, we used the software GetData Graph Digitizer to evaluate their result graph to get an average and SD. We used Review Manager 5.3 (RevMan), 2014) for all of our analyses.

## Results

### Studies selection

**Figure 1:**
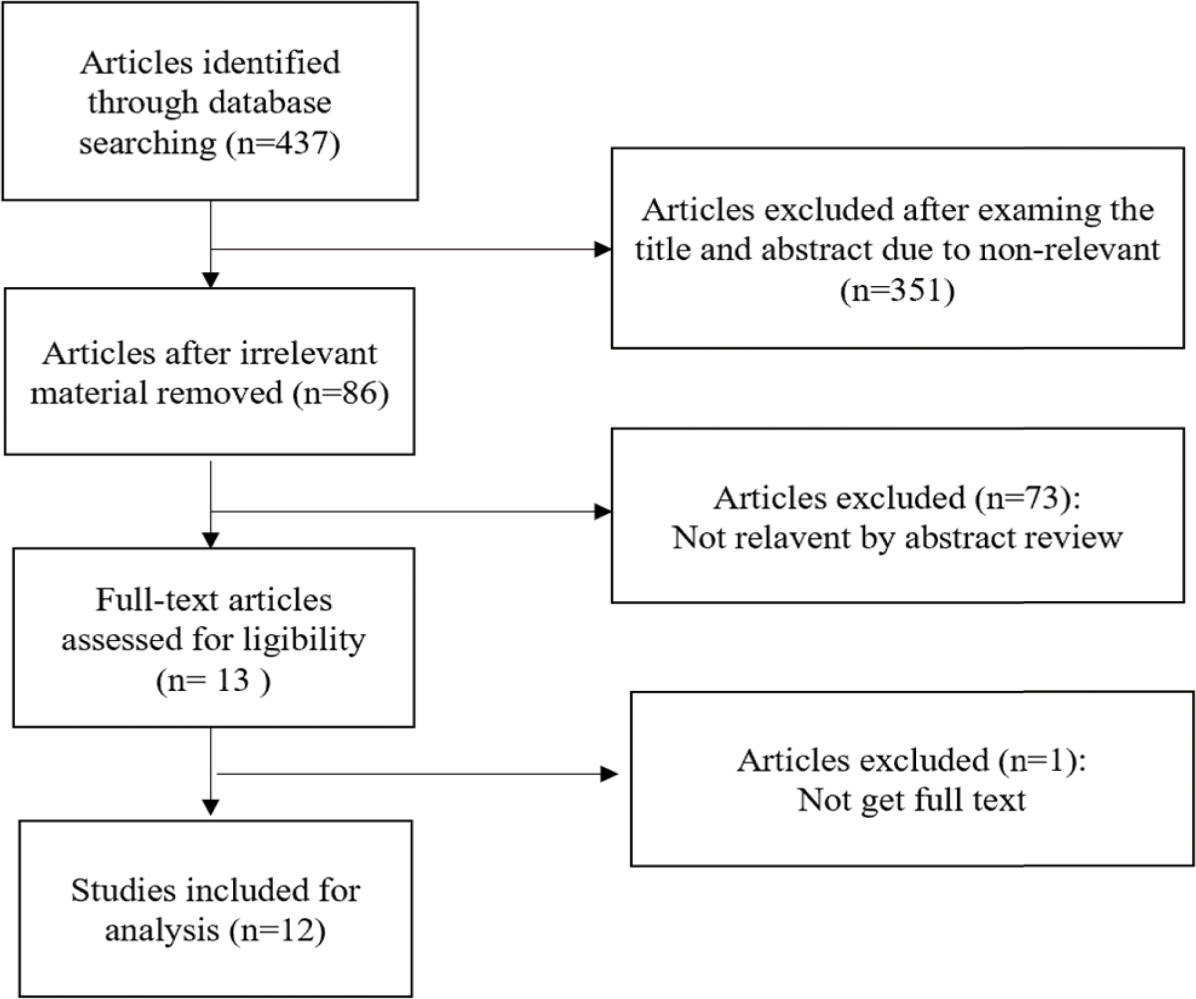
Flow diagram of the literature search and screening process.

The steps of article retrieval filtering are shown in **Figure 1**. We identified 437 articles from the five databases and the bibliographies of relevant articles. By reading the title and abstract of the article, we got 86 articles after eliminating irrelevant articles. After excluding papers not shown in full, duplicates, letters, case studies and those whose themes did not match the criteria of this study, 12 articles remained (**Figure 1**). The two researchers who screened the articles had a high degree of agreement on inclusion and exclusion (Kappa index >96%).

### Included studies characteristics

We selected 12 studies conducted in China. These studies were published between 2018 and 2023 (**Table 1**).

The levels of inflammatory markers, such as TNF-α, decreased in all the studies in which TNF-α was analyzed (15, 16, 19–21, 23, 26). IL-8 levels decreased in three studies (15, 16, 21). In three studies, there were no differences in IL-6 compared to that in the control group (22, 23, 26), but in two other studies (19, 20), there was an improvement in this marker. IL-4 decreased in three studies in which it was analyzed (17, 24, 26), but there were no differences in one study (Wei et al, 2023) (22). Oxidative stress, shown by the MDA levels, was lower in the high-concentration hydrogen group in every study in which it was analyzed (15–17, 23, 26). SOD levels were greater in the high-concentration hydrogen group in every study in which it was (17, 21, 25). ROS decreased in all the studies in which ROS were analyzed (20, 21).

### Data synthesis

In the literature we searched, most of the studies assessed the expression levels of different markers, which made it impossible to conduct a uniform meta-analysis of all the literature, so we conducted a subgroup analysis of the consistent results in some of the literature. Among them, the anti-inflammatory effect of high concentration hydrogen was evaluated using IL-1β, IL-4, IL-8, IL-6 and TNF-α as inflammatory mediators, and the antioxidant effect was evaluated using MDA, SOD and ROS markers.

**Figure 2:**
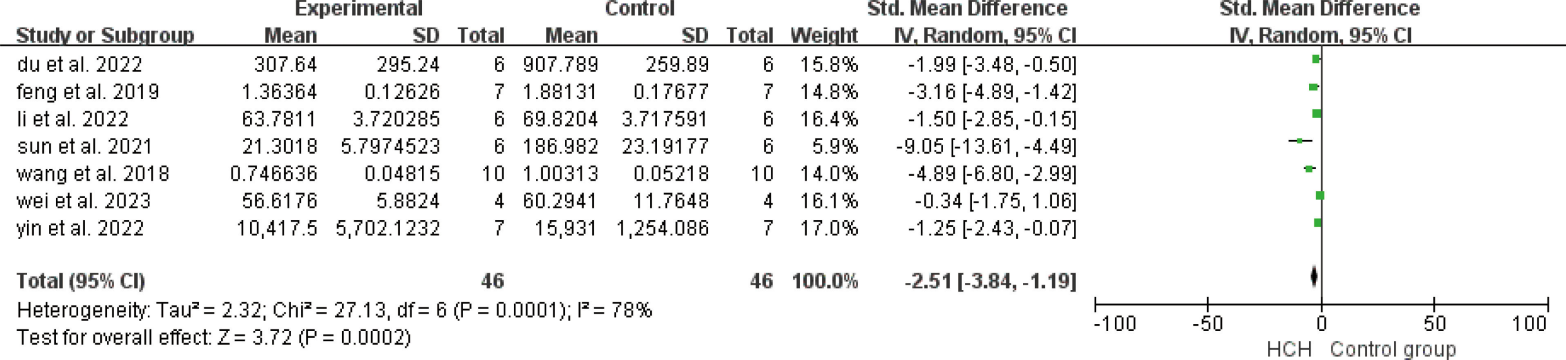
Meta-analysis of IL-1β differences—the HCH group versus the control group.

**Figure 3:**
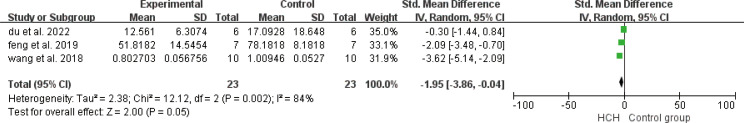
Meta-analysis of IL-8 differences—the HCH group versus the control group.

**Figure 4:**
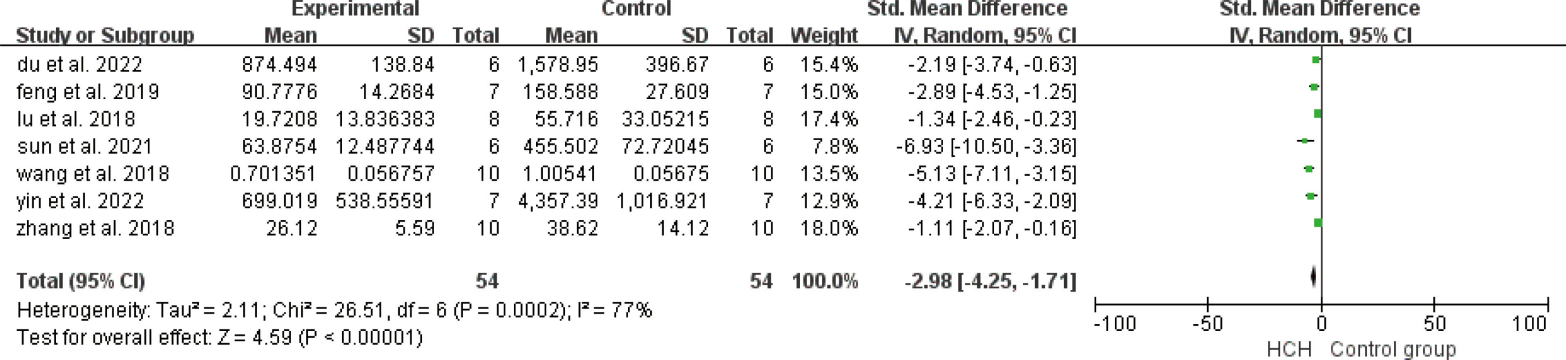
Meta-analysis of TNF-α differences—the HCH group versus the control group.

**Figure 5:**
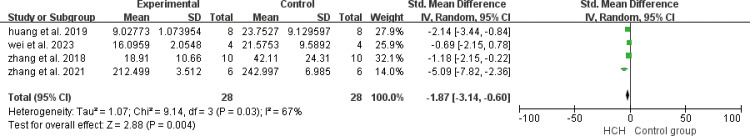
Meta-analysis of IL-4 differences—HCH versus control group.

**Figure 6:**
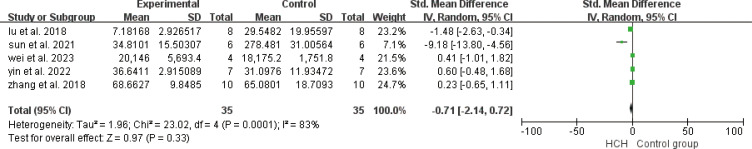
Meta-analysis of IL-6 differences—the HCH group versus the control group.

From **Figure 2**, we can see the protective effect of high concentration of hydrogen on the reduction in IL-1β (SMD = −2.51, 95% CI −3.84 to −1.19, P < 0.005). The I^2^ was 78% and P = 0.0001, indicating that there is high heterogeneity in all studies of IL-1β (**Figure 2**). In order to reduce heterogeneity, we excluded low-quality literature and left two high-quality literature (du et al, 2022; feng et al, 2019) (15, 16). I^2^ < 50%, the fixed-effect model was used to analyze the results, which showed little difference from the original results and that means good stability. From **Figure 3**, we can see the positive effect of high concentration of hydrogen on the reduction in IL-8 (SMD = −1.95, 95% CI −3.86 to −0.04, P = 0.05). The I^2^ was 84% and P = 0.002, indicating that there is high heterogeneity in all studies of IL-8 (**Figure 3**). In order to reduce the heterogeneity, the group with the smallest sample size was excluded (15), and the heterogeneity was reduced to 52%, which still had a significant difference. However, the heterogeneity of retained high-quality literature is still high, and the results are not significant and the results are poor in stability. From **Figure 4**, we can see the protective effect of HCH on the reduction in TNF-α (SMD = −2.98, 95% CI −4.25 to −1.71, P < 0.005). The I^2^ = 77% and P = 0.0002, indicating that there is high heterogeneity in all studies of TNF-α (**Figure 4**). In order to reduce heterogeneity, we retained high-quality literature for analysis (15, 16, 26), and the heterogeneity became smaller, I^2^ < 50%, and the results still had significant differences and they have good stability. From **Figure 5**, we can see the positive effect on the reduction in IL-4 (SMD = −1.87, 95% CI −3.14 to -0.6, P < 0.05. The I^2^ = 67% and P = 0.03, indicating that there is high heterogeneity in all studies of IL- 4 (**Figure 5**). In order to reduce heterogeneity, studies with a small sample size were eliminated (22, 24), and the heterogeneity became smaller with I^2^ < 50%, indicating little difference in results and have good stability. From **Figure 6**, we can see there was no effect on IL-6 (SMD = −0.71, 95% CI −2.14 to 0.72, P = 0.33). The I^2^ = 83% and P = 0.0001, indicating that there is high heterogeneity in all studies of IL-6 (**Figure 6**). To reduce heterogeneity, we assessed the quality of the literature, but there was only one high-quality literature (zhang et al, 2018) (26), and the heterogeneity was high regardless of the group, and there was no significant difference in the results.

**Figure 7:**
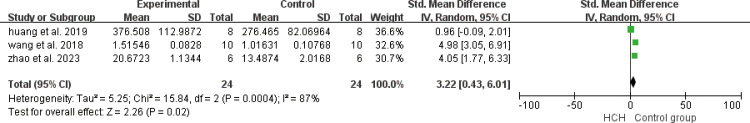
Meta-analysis of superoxide dismutase (SOD) differences—in the HCH group versus the control group.

**Figure 8:**
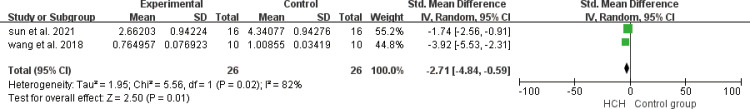
Meta-analysis of reactive oxygen species (ROS) differences—the HCH group versus the control group.

**Figure 9:**
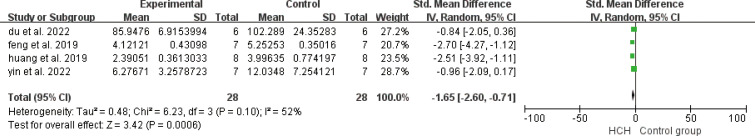
Meta-analysis of malondialdehyde (MDA) differences—in the HCH group versus the control group.

From **Figure 7**, we can see the protective effect of HCH on SOD (SMD = 3.22, 95% CI 0.43 to 6.01, P < 0.05). The I^2^ = 87% and P = 0.0004, indicating that there is high heterogeneity in all studies of SOD (**Figure 7**). In order to reduce the heterogeneity, the minimum SMD was removed for analysis (huang et al, 2019) (17), and the heterogeneity was reduced with I^2^ < 50%. The fixed-effect model was used for analysis, and the results showed no significant difference and have good stability. From **Figure 8**, we can see the positive effect on the reduction in ROS (SMD = −2.71, 95% CI −4.84 to −0.59, P < 0.05). The I^2^ = 82% and P = 0.01, indicating that there is high heterogeneity in all studies of ROS (**Figure 8**). However, there were only two literatures in this group, which could not narrow the heterogeneity for subgroup analysis. From **Figure 9**, we can see the positive effect on the reduction in MDA (SMD = −1.65, 95% CI −2.60 to -0.71, P < 0.05). The I^2^ = 52% and P = 0.10, indicating that there is high heterogeneity in all studies of MDA (**Figure 9**). In order to reduce the heterogeneity, we conducted subgroup analysis of the experimental animal with mouse, the group whose experimental animals were rats was excluded (feng et al, 2019) and the results showed that the heterogeneity was reduced (16), I^2^ < 50%, but the results were not different and the stability was good.

### Risk of bias

**Table 2** summarizes the risk of bias of the 12 studies based on the Systematic Review Centre for Laboratory Animal Experimentation (SYRCLES) risk of bias tool. In Sequence generation, only one study had a low risk (25), they described using a random number table in the literature, while the other studies had unclear risk, there is no evidence how random sequence produced. For baseline characteristics, two studies had low risk (17, 21), they conducted baseline measurement in the article, while others did not describe and had unknown risk. The risk of allocation concealment in all studies is unknown, they all not specify whether there is allocation concealment. Almost all studies showed a low risk in performance bias, they described the same feeding environment and the same administration conditions. For the random outcome assessment, there were five studies with high risk (15, 16, 21, 24, 26), because that did not describe using a random number table to choose experimental animals and the remaining risks were unclear. Meanwhile, six studies showed low risk in detection bias of blinding (15–17, 21, 24, 26), because almost all study used all animal’s results in the outcome that indicate there is no detection bias. There were six studies with low risk in incomplete outcome data (15–17, 21, 24, 26), because all animals were absorbed in the outcome. The rest with high risk (18–20, 22, 23, 25), because they did not describe how to deal with missing data. In reporting bias and other bias, all studies showed low risk. All the data described in method has been reported in results and there is no drug sharing and undue influence from funders. So, they all in low risk.

**Table 2:** SYRACLE’S risk of bias tool for the interventional studies of HCH.

First author, year	Selection bias			Performance bias		Detection bias		Attrition bias	Reporting bias	Other bias
	Sequence generation	Baseline characteristics	Allocation concealment	Random housing	Blinding	Random outcome assessment	Blinding	Incomplete outcome data	Selective outcome reporting	Other sources of bias
du, 2022 (15)	?	?	?	+	+	–	+	+	+	+
feng, 2019 (16)	?	?	?	+	+	–	+	+	+	+
huang, 2019 (17)	?	+	?	+	+	?	+	+	+	+
Li, 2022 (18)	?	?	?	+	?	?	?	–	+	+
Lu, 2018 (19)	?	?	?	+	+	?	?	–	+	+
sun, 2021 (20)	?	?	?	+	+	?	?	–	+	+
wang, 2018 (21)	?	+	?	?	?	–	+	+	+	+
Wei, 2023 (22)	?	?	?	+	?	?	?	–	+	+
Yin, 2022 (23)	?	?	?	+	?	?	?	–	+	+
zhang, 2018	?	?	?	+	+	–	+	+	+	+
zhang, 2021 (24)	?	?	?	+	+	–	+	+	+	+
zhao, 2023 (25)	+	?	?	+	+	?	?	–	+	+

+: Low risk of bias ‘?’: not mentioned clearly -: high risk of bias.

‘Other bias’ includes the possibility of contamination/pooling drugs, inappropriate influence of funders, and new animals added to the control and experimental groups to replace drop-outs from the original population.

## Discussion

According to Matei et al. (27), the therapeutic potential of hydrogen has received much attention, and researchers have reported that hydrogen has a beneficial effect on a variety of diseases, including lung diseases such as COPD and ALI.

Other studies have shown that HCH has many pharmacological properties, such as antioxidant and anti-inflammatory effects. The anti-inflammatory effect of HCH may be mediated by the regulation of NF-κB (28). The forest plot (**Figure 4**) shows that HCH has a positive effect on reducing TNF-α, and all of the analyzed studies included showed that HCH was able to reduce this inflammatory mediator. According to Gardam (29), TNF-α is a key mediator of the activation and recruitment of inflammatory cells, including polymorphonuclear neutrophils (PMNs) and macrophages. In addition, it can also induce the release of proinflammatory markers and oxidative and nitrosation stress in the lung endothelium (30, 31). According to Carvalho (1), the primary action of IL-8 is to stimulate the migration of immune system cells, mainly neutrophils, to increase the expression of adhesion molecules by endothelial cells. This relationship between IL-8 and neutrophilic stimulation was also observed in studies by Hamahata et al. (32) and Qiu et al. (33). According to Zwahlen et al. (34), IL-8 can also activate polymorphonuclear neutrophils and increase oxidative metabolism. The forest plot (**Figure 3**) shows that HCH has a positive effect on reducing IL-8. Hamahata et al. (32), Laffon et al. (35) and Qiu et al. (33) reported that inflammatory cytokines such as IL-1β play an important role in the occurrence and development of lung injury. In this paper, the effects of HCH on IL-1β were analyzed by making forest plot, and it can be concluded that IL-1β is not only crucial for lung injury, but also can regulate the disease process of lung injury by regulating IL-1β (**Figure 2**). The forest plot shows that for IL-4, HCH can reduce its secretion. According to Kianmehr et al (36), IL-4 has been detected in the BALF and airway biopsies of patients with mild or asymptomatic asthma and COPD.

In addition, according to Lu et al. (19) and Sun et al. (20) reported that HCH can regulate the secretion of TNF-α, IL-6 and HMGB1 to affect inflammation; but our results didn’t support this conclusion. Among the five studies that evaluated the effects of HCH on IL-6 levels, the studies by Wei et al. (22), Yin et al. (23) and Zhang et al. (26) did not discovered significantly change in the level of IL-6, which make it no significant difference in the result of meta-analysis. We hypothesize that the non-significant difference in results may be due to the following reasons: first, the sources of IL-6 measured in literature are different. When detecting IL-6 in BALF in zhang et al (26), there is no significant difference in hydrogen group, but there is a significant difference in serum, but the data processing software cannot obtain this result. Therefore, only IL-6 levels in BALF were analyzed. In addition, yin et al (23) analyzed IL-6 levels in different time periods, each time point showed different therapeutic effects, but in this study, we only analyzed one of the time points, so IL-6 levels are constantly changing in the course of disease and treatment. This time point we chose is not representative of the therapeutic level of hydrogen in IL-6 over the course of treatment.

De Carvalho et al. (37) reported that smoke inhalation can cause lung and systemic lesions, mainly involving inflammatory processes and oxidative stress, in which the oxidative stress mediators include MDA and so on. MDA is an important marker of oxidative stress, and HCH can significantly reduce its production (**Figure 9**). In addition, in a population study, it was found that HCH can significantly reduce the level of MDA in lung disease (38). This study confirms the conclusion that HCH can downregulate the level of oxidative stress in the body in lung disease. The quantification of MDA in biological systems is an important parameter for evaluating cellular oxidative stress and is used to estimate lipid peroxidation in the lung (37, 39). Many studies with hydrogen, which uses this marker to analyze the progression of the inflammatory process, have shown that it has positive effects (40, 41).

ROS include free radicals, such as ·OH, superoxide anion radicals (O_2_· ^−^), and nonfree radical species, such as singlet oxygen (^1^O_2_) and H_2_O_2_ (42). They are generated inside the body by aerobic organisms as a byproduct of energy metabolism through oxidative phosphorylation (43). Normally, there are antioxidant defense systems in cells that protect biological systems from free radical toxicity, such as SOD, catalase (CAT), glutathione peroxidase (GSH-Px), and heme oxygenase-1 (HO-1) (44, 45). The effects of HCH on ROS and SOD were studied in this paper. Positive effects were observed in the included studies.

Hancock et al. (46) states that hydrogen, a well-known antioxidant, has possible positive effects on lung diseases. Its oxidative stress-reducing parameters have been widely studied in several pathologies (47–49).

However, this study has certain limitations. All the included studies were from China, and the application of high concentrations of hydrogen needs to be confirmed by more researchers. In addition, for cases with heterogeneous sources or no significant difference, we analyzed that the therapeutic effect of hydrogen in the indicators of inflammation and oxidative stress changes with time, so there should be studies to explore which stage hydrogen plays the most important role in the occurrence of inflammatory factors or oxidative stress. Or explore whether hydrogen mainly works by acting directly on the lungs or after entering the blood. Finally, most of the published clinical studies on hydrogen in the respiratory system are on HCH, and the detection indicators are limited to the relief of clinical symptoms. More in-depth clinical studies need to be carried out.

## Conclusion

Our results suggest that high concentrations of hydrogen have anti-inflammatory and antioxidant effects in certain inflammatory or oxidative stress mediators. However, at present, there are problems such as small sample size of animal studies or small number of human experiments. A more targeted experimental design would make it possible to more clearly elucidate the relationship between high concentrations of hydrogen and mediators of inflammation and oxidative stress. For now, more high-quality studies are needed to validate these findings. Whether there are more effects on reducing inflammation and oxidation mediators remains to be further elucidated.

## Data Availability

The raw data supporting the conclusions of this article will be made available by the authors, without undue reservation.

## Glossary

**Table:**

ALI	Acute lung injury
H2	Hydrogen
HCH	High concentration hydrogen
BALF	Bronchoalveolar lavage fluid
CAT	Catalase
sirt1	Sirtuin-1
ICAM-1	Intercellular cell adhesion molecule-1
VCAM-1	Vascular Cell Adhesion Protein 1
CAPs	Concentrated ambient particles
MUC5A	Mucin 5AC
8-iso-PGF2α	8-iso-prostaglandin F2α
AhR	Aryl hydrocarbon receptor
PMN	Polymorphonuclear neutrophil
TUNEL	TdT-mediated dUTP Nick-End Labeling
KC	Keratinocyte-derived chemokine
MIP-1α	Macrophage inflammatory protein-1α
MIP-2	Macrophage inflammatory protein-2
MCP-1	Monocyte chemoattractant protein-1
Nrf2	Nuclear factor erythroid-related factor 2
HO-1	Heme oxygenase 1
ASC	Apoptosis-associated speck-like protein containing CARD
GSDM-D	Gasdermin-D
BCL-2	B-cell lymphoma-2
TBI	Traumatic brain injury
ERK	Extracellular regulated protein kinases
ROS	Reactive oxygen species
HE	Hematoxylin and eosin
Ki-67	Protein phosphatase 1, regulatory subunit 105
VEGF	Vascular endothelial growth factor
SMC3	Structural maintenance of chromosomes protein 3
CXCL	Chemokine C-X-C-motif ligand
CCL	Chemokine C-C motif ligand
CSF	Colony stimulating factor
GSH	L-Glutathione
NO	Nitric oxide
TLR	Toll like receptor
MPO	Myeloperoxidase
8-OHdG	8-hydroxy-2 deoxyguanosine
TSLP	Thymic stromal lymphopoietin
ST2	Tumorigenicity 2 receptor
ZO-1	Zona occludens 1
ILC	Innate lymphoid cell
HMGB1	High mobility group box 1 protein
RCR	Mitochondrial Respiratory Control Rate
MMP	Mitochondrial membrane potential
IFNγ	Interferon gamma
IL	Interleukin
LPS	Lipopolysaccharides
MDA	Malondialdehyde
MPO	Myeloperoxidase
NF-κB	Nuclear factor-kappa B
SOD	Superoxide dismutase
TNF-α	Alpha tumor necrosis factor
CO	Carbon monoxide
SMD	Standardized mean difference
CIs	Confidence intervals
SDs	Standard Deviation
SYRCLES’s	Systematic Review Centre for Laboratory Animal Experimentation
PMNs	Polymorphonuclear neutrophils
